# Youth Participatory Action Research (YPAR) in Action: a Case Study of Addressing Youth Violence and Social Inequities in New Orleans

**DOI:** 10.1007/s11121-025-01869-w

**Published:** 2025-12-27

**Authors:** Micaiah A. Lugo, Rae Stevenson, Andrea L. DaViera, Manuel A. Ocasio, Timothy Craft, Jade Lewis, Ana Fujisaki, Julia Fleckman, Katherine P. Theall, Samantha Francois

**Affiliations:** 1 Center for Youth Equity, Tulane University, New Orleans, LA USA; 2 Violence Prevention Institute, Tulane University, New Orleans, LA USA; 3 Department of Education, Clark University, Worcester, MA USA; 4 Department of Pediatrics, Tulane University School of Medicine, New Orleans, LA USA; 5 Department of Social, Behavioral, and Population Sciences, School of Public Health and Tropical Medicine, Celia Scott Weatherhead, Tulane University, New Orleans, LA USA; 6 Department of Psychology, Clark University, Worcester, MA USA

**Keywords:** Youth Participatory Action Research (YPAR), Violence prevention, Black youth, Sociopolitical development, Positive youth development

## Abstract

Black youth in New Orleans experience some of the highest rates of violence in the US. Although Youth Participatory Action Research (YPAR) is recognized as an effective tool for empowering young people and fostering social change, its application in violence prevention remains an area worthy of further application. This case study presents a YPAR-based program designed to address structural issues, including racism, discrimination, and economic disinvestment, that contribute to youth violence. The Enrichment to Empowerment (E2E) program provides a platform for youth to advocate for systemic change in their communities. This paper offers actionable insights into equity-centered approaches for violence prevention, highlighting the potential for YPAR programs as a strategy for addressing complex, community-driven issues through youth-led initiatives.

## Introduction

In the USA, youth violence is a significant issue; homicide has been among the leading causes of death among youth for decades (Centers for Disease Control and Prevention [CDC], 2023). Since the 1980 s, homicide has consistently ranked among the top three to five causes of death for youth aged 10–24 and in the top 2 for Black youth (CDC, 2023). Nowhere is this disparity more evident than in the Gulf South, with Louisiana currently holding the highest homicide rate in the nation (CDC, 2023). Within Louisiana, New Orleans consistently ranks among the nation’s most violent cities (Louisiana Department of Health, 2022).

In many ways, New Orleans is a microcosm of the structural inequities that fuel and perpetuate youth violence, making it a critical site for examining youth violence prevention efforts. Economic disinvestment, a fragmented, market-based education system, and policies that criminalize youth, such as the automatic processing in adult court for 17-year-olds (Louisiana State Legislature, 2024), have deepened racial disparities and exacerbated the conditions that drive violence (Buchanan et al., 2021; Webster, 2024). The compounded effects of these inequities, worsened by disaster recovery policies and a lack of mental healthcare infrastructure, have left many young people without the resources or support needed to navigate trauma (Rosenberg et al., 2022). Further, intervention strategies that rely on punitive responses such as intensive juvenile probation programs (Lane et al., 2005), mandated community treatment blending care and control (Skeem et al., 2007), and behavior management models like Positive Behavioral Interventions and Supports (PBIS) within juvenile facilities (Boden et al., 2020) fail to address these underlying causes and often perpetuate the very conditions they aim to resolve.

Recent scholarship has advanced the field’s recognition of young people as experts in articulating systemic harm and deepening understanding of how structural violence—including racism, housing instability, and inadequate access to care—shapes youth mental health and exposure to violence. Across a growing body of youth-led and participatory research, young people have examined the effects of community violence, discrimination, and systemic neglect, while proposing alternatives rooted in healing and collective action (Harden et al., 2014; Villa et al., 2018). For example, Fisher et al. (2025) employed youth participatory action research (YPAR) to explore how intersecting systems of oppression shape mental health among marginalized youth, offering policy-oriented solutions grounded in lived experience. While such studies have broadened the field’s understanding of structural violence and emphasized the value of youth knowledge, few position YPAR itself as the central mechanism of intervention.

This paper presents a detailed case study of the Enrichment to Empowerment (E2E) program, a youth violence prevention initiative in New Orleans that utilized YPAR as its core intervention strategy. Situated within Tulane University’s Center for Youth Equity and part of the CDC-supported Youth Violence Prevention Center (YVPC) network, E2E leverages YPAR to address the structural determinants of youth violence, including systemic racism, economic marginalization, and policy neglect, through a multi-phase model grounded in sociopolitical development, trauma-responsive pedagogy, and youth-led public action.

While YPAR is widely recognized as an effective method for fostering critical consciousness and civic engagement among youth (Anyon, et al., 2018), its direct application as a violence prevention intervention remains underexplored, with key implementation details often overlooked. We aim to address this gap by presenting a case study of E2E’s use of YPAR as a strategy for violence prevention. This paper contributes to the field by offering a practice-based account of how YPAR can function as a youth violence prevention intervention that builds individual capacity, fosters healing, and drives structural change. By outlining the program’s design, core principles, and on-the-ground practices, we demonstrate how YPAR can be utilized to confront systemic inequities and position youth as central actors in shaping structural responses to violence in New Orleans and beyond.

## Conceptual Framework

### Sociopolitical Development and Positive Youth Development

The conceptual Framework for E2E was informed by Sociopolitical Development (SPD) and Positive Youth Development (PYD). SPD and PYD offer strengths-based alternatives to deficit-oriented models of youth violence prevention (Lerner et al., 2002; Watts et al., 1999, 2003), similar to Ginwright and Cammarota’s (2002) social justice youth development framework. SPD, grounded in Paulo Freire’s (1970) concept of critical consciousness, focuses on how marginalized youth develop awareness and collective agency to challenge systemic inequities (Diemer et al., 2016; Watts et al., 2003). It reframes violence as a structural issue rather than an individual failing and positions youth as active agents of change. SPD is complemented by PYD, a strengths-based model that prioritizes youth agency, the development of socioemotional skills, and supportive environments (Gonzalez et al., 2020; Lerner et al., 2002). Together, these frameworks promote cognitive, socioemotional, and behavioral development through mentorship, leadership, academic and vocational enrichment, and civic engagement. It emphasizes protective factors and holistic development, ensuring that youth are not merely intervention recipients but active participants in shaping their communities. These frameworks reflect Social Justice Youth Development which also equips young people with tools to navigate, resist, and transform oppression (Ginwright & Cammarota, 2002). Integrating SPD and PYD shifts violence prevention from reactive intervention to proactive empowerment. In New Orleans, where Black youth face structural racism and economic disinvestment (Center for Urban & Racial Equity, 2020), this combined approach fosters both critical consciousness and leadership. SPD helps youth understand the systemic roots of violence, such as criminalization, school pushout, and economic marginalization, while PYD provides tools to build networks of support and drive social change. Drawing from these frameworks, the E2E project empowered youth to identify root causes of violence and take action through group discussions, research, and advocacy.

### YPAR as a Transformative Tool

YPAR translates the insights of SPD and PYD into a concrete strategy for youth-led change (Diemer et al., 2017). YPAR is a critical research methodology (Caraballo et al., 2017; Filipiak, 2022) that positions young people as co-researchers, drawing on their lived experiences to investigate and transform conditions affecting their lives (Cammarota & Fine, 2008; Rodriguez & Brown, 2009). Rooted in co-creation and collective action, YPAR functions as both research and political intervention, equipping youth with analytical and organizing tools to challenge structural inequities. Unlike traditional research approaches, YPAR operates at the intersection of knowledge production and movement-building, turning research into a structural intervention.

While some studies have examined youth voice (Banyard et al., 2022; Waterman et al., 2021) and community-based participatory research in violence prevention, most have been adult-centered and few evaluated YPAR’s direct role in disrupting cycles of harm (Call-Cummings et al., 2023; Villa et al., 2018). Further, existing research highlights YPAR’s ability to foster leadership and critical consciousness (Anyon et al., 2018; Mirra et al., 2015) but rarely examines YPAR’s impact on reducing or preventing youth violence. This gap reflects a broader reluctance to recognize Black youth in communities most affected by structural violence as knowledge producers and key actors in shaping public safety. Framing of youth as either at-risk or in need of intervention erases the ways they already engage in safety-making and resistance.

Within E2E, YPAR was utilized to engage youth in qualitative research, policy analysis, and advocacy campaigns to address structural determinants of community violence on both a systemic and interpersonal level. This participatory process fostered ownership of solutions and positioned youth as credible experts. By equipping youth to question and analyze systemic oppression, YPAR cultivated the critical consciousness central to SPD (Kornbluh et al., 2015). YPAR was used to activate this consciousness by helping youth recognize violence as a product of oppression and something that can be prevented through collective action (Allison et al., 2011; Nation et al., 2021). At the same time, E2E embodied PYD by strengthening youth cognitive and socioemotional skills, fostering mentorship, and creating opportunities for leadership and empowerment (Harden et al., 2014; Iwasaki et al., 2014). Further, teaching community organizing strategies reinforced these efforts, ensuring that youth were not passive recipients but active participants in shaping community outcomes (Zimmerman et al., 2011).

## Methods

### Guiding Principles of E2E

E2E was facilitated and coordinated by adult program staff, while the approach was shaped and informed by youth. The E2E program was grounded in four guiding principles that informed every aspect of its design and implementation: anti-racist/anti-oppression frameworks, centering joy and play, creating safe spaces and building community, and power-sharing and youth autonomy. These principles ensured that youth were empowered to research systemic violence and co-create solutions within a supportive and affirming environment. Each principle is further detailed in the following sections.

#### Anti-racist and Anti-oppression Frameworks

Drawing from the political commitments of both SPD and YPAR, our curriculum supported youth in understanding how intersecting systems and identities of race, class, and gender shape violence in their city. Informed by critical race theory (West et al., 1995), Black feminism (Crenshaw, 1989, 1991), and abolitionist pedagogy (Kaba et al., n.d.), we introduced how community violence could be attributed to structural racism and economic disinvestment and provided youth with opportunities to reflect on their own lived experiences. Some activities included participating in tours that taught about the history of urban enslavement in New Orleans and learning about community organizing from local social justice organizations. Through activities and discussion, youth interrogated dominant assumptions about community safety and justice, situating their research within broader sociopolitical contexts. These frameworks laid the groundwork for designing a meaningful project targeting structural inequities.

#### Centering Joy and Play

We define centering joy and play as creating opportunities for fun, building community, fostering meaning-making, and establishing a safe and inclusive space. Research notes that centering joy and play supports collective well-being, affirms cultural identity, and helps sustain engagement in justice work, by building resilience, nurturing imagination, and fostering connection and hope (Ginwright, 2018; Scipio et al., 2025). Play creates opportunity for meaning-making, allowing youth to process and navigate difficult conversations without becoming overwhelmed. Furthermore, joy serves as an act of resistance (Griffin & Turner, 2021; Hall & Steele, 2024) against oppression, reinforcing the humanity and agency of those involved in liberation struggles.

Throughout the program, joy and play were intentionally prioritized to maintain motivation and engagement. Each session began with sharing good things happening in participants’ lives, creating a positive and supportive atmosphere. Activities were playful and interactive, incorporating gamification, pop culture, memes, icebreakers, drama exercises, movement breaks, and field trips. When activities naturally evolved into moments of laughter and lightheartedness, we embraced these moments rather than rushing. These instances allowed the group to bond and create shared memories, reinforcing the value of joy in learning.

#### Creating Safe Spaces and Building Community

Creating a safe space for youth was foundational to fostering trust and authentic dialogue. Within youth development literature, “safe space” is often conceptualized as an environment where individuals feel secure expressing themselves without fear of judgment or harm (Arao & Clemens, 2013). Rather than a static concept, safety in dialogue requires continuous negotiation and active facilitation to ensure inclusivity and responsiveness to diverse experiences. We sought to create a space where youth could engage authentically and critically with complex social issues. Facilitators modeled vulnerability by sharing first during discussions and leading improvisation exercises. Regular “getting to know you” activities, such as personal bingo, further promoted inclusivity. Allowing participants to express themselves freely, including their style, individual preferences (e.g., listening to music through headphones), and contributions to shared playlists, encouraged authenticity and comfort.

We also made space for emotional processing, especially as youth explored experiences of systemic oppression and community violence. Facilitators regularly invited participants to reflect on what they learned, how it made them feel, and what it made them think about. Youth were encouraged to draw connections to their lived experience, name their emotions, and take breaks when the topics felt too heavy. Throughout the program, we validated youth’s feelings, affirming their hurt, surprise, denial, joy, and confusion that surfaced during discussions. We also validated the diversity in lived experiences and held space to acknowledge that multiple realities co-exist and some are not universal. Allowing space for emotional processing during the learning process kept the youth grounded in their why and offered space for healing.

Community-building activities allowed youth to share experiences, offer support, and make sense of their worlds. When external events impacted participants, the group provided a vital support system. Youth shared resources, advice, and genuine camaraderie. While disagreements occasionally arose, the community agreements they co-created fostered respectful conflict resolution. Participants resolved conflicts on their own. After conflict, program staff reminded youth about being mindful of how their word choice may affect others and respecting differences. We reinforced the idea that the group may not always reach consensus, but community members could disagree and still remain in solidarity. The youth navigated differences through compromise or agreeing to disagree, ensuring that respect and support remained central to their interactions. This approach enabled youth to engage deeply with challenging topics, build meaningful connections, and cultivate a shared sense of purpose.

#### Power-Sharing and Youth Autonomy

Youth may lack certain tools or resources to fully actualize their visions. This is where adult collaborators and power-sharing processes played a crucial role. Staff carefully balanced stepping back to let youth lead and stepping in to provide support. Tasks and responsibilities were designed to be developmentally appropriate, accessible, and responsive to youth needs (Ballonoff et al., 2019). Youth decision-making was a foundational principle that guided the course of the project. Their input shaped guest speaker invitations, session content, and new activities. They chose meals for group lunches, selected preferred icebreakers and movement breaks, and could take personal breaks when needed. They were encouraged to speak openly, whether reflecting on personal experiences like what it means to be an introverted Black girl. These moments of choice, dialogue, and reflection strengthened their confidence and leadership and cultivated a collective sense of ownership. These practices reflect key components of Critical Youth Empowerment (CYE), which emphasizes youth-determined and youth-directed activities, authentic opportunities to practice leadership, and equitable power-sharing between youth and adults (Jennings et al., 2006).

### E2E Participants and Recruitment

Youth were recruited through various platforms, including social media, community events, parks, playgrounds, schools, and direct outreach to youth-serving organizations. These efforts resulted in 50 applicants; 23 youth (ages 14–20) were selected. They included 9th graders through college first-years. We recruited youth from middle adolescence (14–15 years old) to emerging adulthood (19–20 years old) because younger teenagers are not typically afforded leadership development opportunities, and emerging adults are often more likely to have exposures to violence (Dugan & Leonette, 2021).

The E2E cohort consisted of 19 female and four male and 21 self-identified as Black or African American, two as white. We did not have recruitment targets related to youth race/ethnicity or gender; the racial/ethnic make-up of the youth participants reflects the composition of New Orleans youth where 71% identify as Black and 22% identify as white (NOLA Kids Data Book, 2020). Two youth reported having past research or activism experience.

### E2E Program Design

Program components and curricula were developmentally tailored using scaffolded skill-building, developmentally appropriate and action-oriented engagement, social-emotional supports, and flexible and youth-led structure (Ballonoff et al., 2019, Ballonoff et al., 2024). These adaptations fostered peer-to-peer learning and coaching that transcended age and stage of development.

#### Program Staff Positionality Statement

Research is never neutral; it is fundamentally shaped by who we are, our lived experiences, identities, values, and social positions. As a research team grounded in community-based, justice-oriented work, we acknowledge the perspectives we brought to this project and how they shaped our approach to youth violence prevention. Many of us come from historically marginalized communities, and our collective identities, encompassing experiences of race, gender, sexuality, geography, and class, inform a shared sense of responsibility toward justice-driven research. Although these positionalities may have introduced certain biases, they deepened our accountability, urgency, and responsiveness throughout the study. Our theoretical orientation, combined with lived experience in the region, deeply informed our project framework and attentiveness to structural context.

Our diverse perspectives made us attuned to power dynamics, cultural responsiveness, and the importance of creating space for youth leadership. We were committed to making the process flexible, relational, and rooted in care, recognizing that preventing violence involves not just data collection, but healing, trust-building, and collective transformation. What emerged was not just a research project, but a collaborative process that honored the complexity of youth experiences while working toward meaningful change in their communities.

### E2E Program Implementation

To ensure a structured and intentional approach rooted in YPAR best practices, the program was divided into three phases implemented from Summer 2023 through Spring 2025: *Phase 1—the YPAR summer academy, Phase 2—research and action planning, and Phase 3—the action campaign*. Table 1 offers a broad breakdown of each phase’s structure and core activities. The phased approach reflects the iterative nature of YPAR, allowing youth to progressively develop skills, deepen their critical consciousness, and refine their advocacy strategies. Drawing from established models of participatory research with youth (Cammarota & Fine, 2008; Kornbluh et al., 2015), each phase was designed to scaffold youth learning and support engagement that was responsive to their developmental stage, interests, and availability while maintaining a trajectory toward actionable change.

**Table 1:** Structure of the E2E program

Topics covered	Activities and community involvement	Desired outcome
**Phase 1. Summer YPAR academy**		
▪ YPAR and arts-based research methods ▪ Qualitative and quantitative research methods; mixed methods ▪ Power dynamics, historical and systemic racism ▪ Community issues and asset mapping	▪ Team-building exercises ▪ Field trips ▪ Guest speaker sessions ▪ Group discussions ▪ “Ideal New Orleans” visioning ▪ Comparative analysis of local and ideal neighborhoods	▪ Establish a foundational understanding of YPAR as a methodological and epistemological framework ▪ To foster a critical reflection of the structural determinants of youth violence
**Phase 2. Research and action planning**		
▪ Root causes of violence ▪ Community-based interview techniques ▪ Case studies of successful arts-based interventions	▪ Socratic seminars ▪ Interview practice ▪ Story mapping exercises ▪ Community Story Collection ▪ Community fieldwork	▪ Gather narratives from community members to better understand systemic issues ▪ Analyze collected data to inform advocacy strategies
**Phase 3. Action campaign**		
▪ Power and local governance ▪ Meeting with stakeholders ▪ Public safety ▪ Storytelling as civic practice ▪ Grassroots organizing ▪ Policy advocacy and public speaking	▪ Film production and editing ▪ Petition development ▪ Community asset mapping ▪ Film screenings and public forums ▪ Guest speakers and panel discussions	▪ Implement advocacy initiatives based on research findings ▪ Advocate for youth-centered policy and resource allocation in New Orleans

#### Phase 1: Summer YPAR Academy

The summer academy’s goals included: (1) building a community among the youth cohort participating, (2) understanding the root causes of violence in New Orleans, and (3) establishing foundational knowledge of YPAR, enabling youth to create a research project targeting community violence. The summer academy spanned 4 weeks, meeting 5 days a week from 10 a.m. to 3 p.m. We emphasized that the group and project belonged to the youth, with adults serving as facilitators and supporters. To reinforce this, an early activity had youth envision their ideal New Orleans, using it as a foundation for their research and to develop a vision for change created by youth. Youth set collective goals and made key decisions about the project’s direction. Discussions centered on systemic racism, access to resources, and intergenerational lived experiences. Field trips to historical sites like the New Orleans Historic Collection and an urban enslavement tour highlighted the city’s long history of oppression and resistance, providing crucial historical context that connected past movements to present-day struggles. Throughout the academy, youth were trained in research methodologies, including YPAR and arts-based research, and qualitative approaches. These sessions were primarily taught by Senior Program Manager M.L., with additional facilitation by community partner Beloved Community and E2E Director S.F. Together, they ensured sessions were engaging, developmentally appropriate, accessible, and grounded in a youth-centered framework (Ballonoff et al., 2019, Ballonof et al., 2024). Youth co-developed a mixed-methods research project, incorporating surveys and interviews to examine violence in their communities.

#### Phase 2: Research and Action Planning

The research and action planning phase of E2E consisted of ten consecutive Saturday sessions, culminating in a showcase of the youth’s research findings and call to action. One of our main priorities was shifting youth perspectives from individual responsibility for violence to understanding systemic causes. Lessons led by M.L. and R.S. explored structural racism and historical inequities through storytelling and creative expression. For example, youth watched spoken word performances that critiqued various forms of oppression in America, which then served as the foundation for Socratic seminars. These sessions fostered meaningful dialogue around both personal and systemic experiences with violence, helping participants explore the distinctions between interpersonal and structural violence and how they are interconnected (Polite & Adams, 1997). Field trips deepened these discussions, including visiting an exhibit that connected history to the present through powerful artwork depicting the treatment of Black lives. Youth also engaged in the creative process, envisioning their own radical dreams and expressing them through artwork led by local artists. These experiences emphasized the power of collective action and encouraged youth to view their advocacy as part of a broader historical struggle.

After exploring several youth-led projects, the group shifted data collection methods, selecting documentary filmmaking as their approach. This shift allowed for more creativity that sustained youth interest and offered a meaningful outlet for self-expression. They eliminated the survey component of their research and refined their interview guide to collect qualitative data through filmed interviews. Youth practiced interviewing one another before conducting interviews with community members and leaders. To structure their documentary, they developed a storyboard that balanced a realistic portrayal of systemic violence with an optimistic vision for change. One youth member took the lead on editing with the support of program staff. Multiple drafts of the documentary were reviewed collaboratively, ensuring the final product reflected the collective vision. The youth titled their documentary, *This is New Orleans: Stories of Truth and Hope* (https://youtu.be/ZwmBNTqwWtY?si=pcBvhTgT0fHoKmyT). The initial screening was held for family and friends, including a panel discussion where youth gathered audience feedback. They used this feedback to re-edit the documentary before presenting it to a wider audience.

#### Phase 3: Action Campaign

The final phase of E2E included eight weekly sessions and a public screening of the documentary for the broader New Orleans community. This phase focused on civic engagement, advocacy, and sociopolitical development. M.L. led the overall session planning and facilitated activities on applying advocacy tools to youth-led social action. The Louisiana Public Health Institute (LPHI) led sessions on local governance (e.g., how to identify decision-makers, communicating effectively with elected officials) and storytelling in movement-building, using the documentary to amplify their message. Sessions covering advocacy tactics such as petitions, phone zaps, and letter-writing campaigns were led by Eyes on Surveillance, a local grassroots coalition. The youth refined their call to action based on feedback from the original audience to focus their advocacy on increased, sustained funding for youth programs that promote positive youth sociopolitical development. They created a petition urging the New Orleans City Council to invest in youth programming and invited council members to their showcase. The final showcase, featuring the re-edited documentary, was attended by over 70 community members. Youth presented their call to action and provided tangible ways for attendees to support their advocacy efforts. This event marked the culmination of their research and activism.

Since the launch of the E2E social action campaign, the youth have participated in six community events to showcase their documentary. These events included screenings during Youth Justice Action Month and at a youth gun buyback event. Additional showcases were held in two historically underserved neighborhoods. Each event received strong community support and created opportunities for dialogue with city officials and other influential stakeholders. Notable attendees included the Orleans Parish Sheriff, City Council members, the Criminal Justice commissioner, representatives from the mayor’s office, and leaders of local nonprofit organizations working on public safety and equity.

Events held in more central areas of New Orleans drew larger audiences. In contrast, screenings hosted in more geographically isolated and under-resourced neighborhoods, despite high registration numbers, experienced lower turnout. Timing may have also played a role, as some events occurred during busier times of the year. These patterns have implications for future campaigns, including the potential benefits of condensing the screening schedule and further exploring structural barriers to participation in marginalized areas. Nevertheless, the community showcases collectively attracted over 230 attendees, further amplifying the youth’s message and advocacy efforts. The documentary is now available on YouTube, with over 300 views, and is being adapted into short videos for social media to broaden its reach.

### Qualitative Methods

#### Youth Voice and Participatory Validation

As part of the broader evaluation of E2E, we conducted five individual semi-structured interviews and two focus groups with youth participants. We explored personal growth, peer dynamics, and perceptions of social change through E2E. A central focus was on the campaign component of E2E, the creation and public screening of *This Is New Orleans (*https://youtu.be/ZwmBNTqwWtY?si=pcBvhTgT0fHoKmyT), and its associated petition. All sessions were facilitated by two trained E2E program staff, with four interviews and both focus groups conducted in person and one interview via Zoom. Interview and focus group guides included prompts such as the following: “Has the social action campaign (the documentary screenings and petition) helped you to understand community action and its connection to change better? If yes, how? If no, why not?” and “Do you feel you have grown personally by being part of E2E?”.

All individual interviews were video recorded with consent. Audio was transcribed verbatim and de-identified.

## Findings

While a comprehensive thematic analysis is forthcoming, preliminary findings are included here to provide illustrative support for such a program. The findings highlight youth-reported growth in communication, emotional maturity, sociopolitical awareness, and collective responsibility.

### Growth in Confidence and Communication

Several participants described significant growth in their ability to speak with others, particularly in public or unfamiliar settings. One youth shared, “I’ve gotten more comfortable with just starting up a conversation with people randomly… From before where I used to hateeven the idea of meeting someone new.” Another reflected on how engaging with diverse peers during the program pushed them to develop new interpersonal skills: “It taught me patience, a lot of patience… I had to learn how to talk to people I didn’t always understand and still work toward the same goal.”

### Perspective-Shifting and Sociopolitical Awareness

Youth also reported deepening their understanding of community issues and the systems that shape them. One participant said, “Participating in E2E… helped me grow and consider more perspectives. Accomplishing something so big, making real change in our community, made me want to do it more.” Another reflected on their evolving sense of responsibility, noting, “I realized I had a greater responsibility for the young people around me and had to be very intentional in the way that I interacted with them.” Separately, a member check was conducted to validate the accuracy of the paper’s representation of youth experience. Youth were invited to review a narrative summary of the paper’s key claims about the program and its outcomes. They were asked to reflect on what felt true to their experience, what might be missing or misrepresented, and what they would want others to know. This process served both as a validity check and as an opportunity to incorporate youth perspectives directly into the manuscript. One youth affirmed, “I like it! It sounds real to me, but I would add that y’all had people come in and teach us about systemic racism and different types of discrimination… This program taught us healthy ways to communicate while also expressing ourselves.” Another noted, “It looks good, there’s nothing I can think of right now that we’re missing, and it’s pretty accurate to what took place.” These reflections confirmed alignment between the paper’s claims and participants’ lived experiences, while also highlighting the importance of naming specific learning moments that supported youth knowledge-building and advocacy skills. Together, these reflections offer a window into the kinds of personal and collective transformation that occurred through E2E.

## Discussion

E2E, as a sociopolitical and positive youth development intervention, revealed several important takeaways regarding flexibility, power-sharing, iterative learning, and community partnerships that are consistent with findings that discuss similar challenges (Abraczinskas et al., 2022; Renick et al., 2024). These lessons are critical for guiding similar projects in the future.

One of the most important lessons learned was the value of flexibility and iterative learning (Dworski-Riggs & Langhout, 2010). While structure and schedules were essential, we quickly recognized the need for buffer time to accommodate the evolving nature of the project and the needs of the youth in the room. When pre-planned lessons failed to resonate, we chose to pivot quickly, often incorporating movement breaks or spontaneous activities to re-engage participants. We also regularly adapted the campaign based on the evolving needs of the youth, moving sessions, adding additional ones, or postponing events to accommodate their schedules and challenges. This flexibility supported deeper learning through revisiting key ideas over time. For example, after discussing acts of resistance, enslavement tours led us to revisit and deepen the conversation around resistance, illustrating the value of recursive engagement. These practices align with prior findings that emphasize the importance of adaptability in community-based youth violence prevention efforts (Allison et al., 2011; Nation et al., 2021).

Our commitment to flexibility was not without its own set of challenges, particularly in balancing youth-led processes with the constraints of external timelines. The structured timeline of the broader initiative required certain milestones to be met, which at times limited the depth of skill-building opportunities for youth. Ideally, a fully youth-led approach would allow for extended learning periods, where youth could develop skills more thoroughly before applying them to project tasks. However, due to time constraints, much of the learning was happening in tandem with implementation. For example, while developing the documentary, youth received lessons on storyboarding and narrative development during the production process. Since there was limited time for full mastery before execution, adults had to fill in gaps where needed. This dynamic echoes the challenges described by Allison et al. (2011), who noted that implementing evidence-based practices in community contexts often entails navigating competing priorities, and that timelines rarely align neatly with the pacing needed for meaningful youth engagement. These challenges resulted in moments when youth engagement was limited not by their interest or ability, but by the necessity of meeting external deadlines. Nonetheless, our ongoing adaptability helped sustain engagement and signaled to youth that their time and circumstances mattered, reinforcing our investment in them as full participants rather than program subjects.

Another significant takeaway was the challenge of power-sharing. Research shows that when adults share power and provide responsive support within developmental relationships, youth motivation and performance can increase (Malorni et al., 2023; Scales et al., 2020). While the goal was to have the project be youth-led, we found that at times, the balance of responsibility needed adjustment. For instance, one youth took the lead on editing the documentary. While their dedication was clear, they lacked the technical skills necessary to complete the project within the time constraints. After receiving feedback from the first screening and discussing it with the youth, they expressed a desire to re-edit the documentary. We responded by partnering with a new community organization whose youth were trained in video editing, bringing in a skilled youth from that program to help bring the youth’s vision to fruition. This experience highlighted the importance of providing adequate support while ensuring youth ownership of the project.

The E2E intervention presents a clear case for the use of YPAR as a viable violence prevention strategy. It challenges the notion that flexibility and unpredictability are liabilities in program design; rather, they are a critical and central piece of an effective intervention. E2E’s success did not stem from a rigid curriculum but from its ability to adapt, centering youth knowledge and responding to their evolving questions. If violence prevention efforts are to be meaningful, they must resist oversimplification, embrace complexity, and trust young people as theorists, analysts, and leaders. E2E was designed with the understanding that youth already have a depth of knowledge about violence, but they often lack the space, tools, and institutional legitimacy to analyze and act on it.

Realizing YPAR’s full potential requires structural commitments. E2E succeeded by prioritizing depth over scale, building sustained, small-group relationships. If institutions and funders are serious about youth-led violence prevention, they must invest accordingly: in facilitators, long-term support, and training. Attempts to impose rigid, quantifiable frameworks reflect neoliberal logics that dilute the model’s transformative power (Abramovitz & Zelnick, 2021; Coleman, 2021). Funders and scholars evaluating the field alike must embrace that YPAR’s flexibility, unpredictability, and activist orientation are not liabilities but central to its effectiveness. Without these qualities, YPAR risks being co-opted into a depoliticized framework, where youth are invited to share their experiences but are denied the resources, authority, and pathways to translate their research into action. Ultimately, YPAR’s broader impact as an intervention lies not only in what they experience, but in what they become: agents of change within systems historically designed to silence them.

### Limitations and Future Directions

While E2E was a successful YPAR initiative, several limitations highlight broader challenges in implementing youth-driven research. These include our focus on New Orleans and its youth, barriers to policy advocacy, constraints in capacity, training, and resource allocation. A limitation of our approach was its focus on New Orleans, where community content and core values can differ greatly from other regions. The experiences and needs of young people are shaped by the unique challenges of their community, and these differences underscore the importance of ensuring initiatives like E2E are culturally relevant and locally tailored. What works in one community may not translate seamlessly to another, and it is essential to consider these local nuances when designing similar projects in other regions.

An additional limitation was that youth were not able to be involved in every step of the advocacy process. We attempted to set up meetings between the youth and city council members multiple times, but these were repeatedly rescheduled and eventually deprioritized in favor of other meetings. Due to the challenges of securing an audience with local government, project staff partnered with local coalitions that had stronger relationships with officials, which helped advance some of the youth’s advocacy goals more effectively.

A key limitation of E2E was the limited capacity of both the research team and community organizations. Initially envisioned as a community-led program, with academic evaluation support, we engaged two community partners during the grant writing phase to shape the structure, incentives, and community needs. However, they could not manage all aspects, specifically recruitment and implementation. When the initial launch saw no youth attendance, the academic team re-launched the program, taking the lead on outreach while partners contributed in more targeted ways such as supplementing curriculum and supporting logistics.

The program operated with a small team, consisting of two members dedicated strictly to research and two involved in facilitation. With such a lean structure, unforeseen circumstances, such as staff illnesses or emergencies, resulted in challenges maintaining consistent programming. In some cases, sessions had to be canceled or facilitated by staff who were not at full capacity. Additionally, curriculum development was a significant challenge due to staffing limitations. While we were able to integrate community partners into specific components of the program, these collaborations were limited in scope due to both organizational constraints and structural barriers related to formalizing university-community partnerships. Staff were often stretched too thin to provide the depth of support needed for a fully enriched YPAR experience.

We recommend future YPAR projects of this scale to allocate time for capacity assessments and intentionally build relationships with community partners before program launch. Universities and funders should anticipate common barriers to entry, such as complex administrative processes, delayed payments, and limited staffing, and develop systems that either reduce these burdens or directly support grassroots organizations in building capacity to engage.

We also recommend a minimum team of four program staff to share facilitation duties, coordinate logistics (e.g., guest speakers, field trips, and meals), and respond effectively to emerging challenges. This structure distributes labor more equitably and helps maintain momentum and consistency. It also allows staff to be more present and responsive, strengthening relationships with youth and community partners alike.

Finally, YPAR initiatives must remain adaptable. Universities that seek to engage in meaningful, community-based research should invest in the infrastructure, flexibility, and trust-building required to support truly collaborative, community-led work.

## Conclusion

The E2E project demonstrates the transformative potential of YPAR as a violence prevention strategy. Moving forward, the lessons learned from E2E offer a roadmap for implementing YPAR as a violence prevention strategy in other contexts, not only as a method, but as an epistemology rooted in shared power, local knowledge, and structural accountability. Policymakers, funders, and community organizations must recognize the value of youth-led research and advocacy, not as tokenistic gestures, but as essential components of systemic change. This requires a commitment to sustained funding, flexible programming, and the creation of meaningful pathways for youth to influence policy and practice.

At the same time, this is a call to action for universities to critically examine and revise institutional policies that hinder community-engaged research. Universities must reduce bureaucratic barriers that prevent grassroots organizations from becoming true partners and provide infrastructure that supports sustained, relational, community-led work. Equally important, academic institutions need to recognize and reward the time-intensive, flexible nature of YPAR and other participatory methods in their tenure and promotion processes. Without these structural changes, researchers face conflicting demands that limit their ability to fully engage with communities and conduct meaningful, youth-centered research. By redistributing power and resources to young people, we can move closer to a future where violence is understood not as an individual failing, but as a structural issue that demands collective, youth-driven solutions.

Ultimately, E2E serves as a powerful reminder that young people are not merely victims or beneficiaries of interventions—they are experts, leaders, and agents of change. When given the tools, platforms, and support to lead, they have the capacity to transform their communities and challenge the systems that perpetuate inequity. The success of E2E is a testament to the resilience, creativity, and vision of the youth of New Orleans. It speaks to the urgent realities throughout the Gulf South and across the world where youth violence is both prevalent and deeply tied to structural inequity. E2E is a call to action for all those committed to building a more just and equitable society.

## Data Availability

The dataset generated or analyzed during the current study is available on request from the authors.

## Declarations

### Statement on the Use of AI Technology

Portions of this manuscript were edited using ChatGPT-4 for copyediting tasks, including sentence clarity, consistency, and grammar. All AI-generated revisions were critically reviewed and selectively incorporated by the authors, who take full responsibility for the content, interpretations, and conclusions presented.
